# Mr. Lawson Tait’s Mistake

**Published:** 1886-08

**Authors:** 


### *MR. LAWSON TAIT'S MISTAKE.*

It is interesting, as well as a source of consolation, to lesser mortals to sit quietly by and witness the mistakes of those to whom we look for something besides error. In a letter written by Mr. Lawson Tait to Dr. R. P. Harris, of Philadelphia, under date of April 16, 1886, on the subject of treatment of extra-uterine pregnancy by faradization, he says: "I have very strong objections to the proposal to treat extra-uterine pregnancy by faradization." After giving his reasons at some length, he concludes with the following: "In the whole course of my life, I have only known one case where the woman has carried an extra-uterine pregnancy for a number of years after the death of the fœtus. We know with perfect certainty all about this case, and for about eighteen years she has carried on the left side a condensed ovum of extra-uterine pregnancy. I doubt very much if there would be found in the whole world three other such cases." * * * *

This latter is a surprising statement, to the refutation of which Dr. Harris brings an interesting collection of statistics, prefaced as follows: "Mr. Tait appears not to be aware of the fact that cases of prolonged ectopia gestation have been comparatively numerous, as witness the following partial record—"

He then gives a *partial* list of twenty-one reported cases where from one to two fœtuses had been carried for periods from fourteen to fifty-six years, more than half of them being for periods over thirty years.

It is strange that Mr. Tait should make so erroneous a statement about anything connected with extra-uterine pregnancy, in which he is an acknowledged master.

